# Low doses of LPS exacerbate the inflammatory response and trigger death on TLR3-primed human monocytes

**DOI:** 10.1038/s41419-018-0520-2

**Published:** 2018-05-02

**Authors:** Marta Monguió-Tortajada, Marcella Franquesa, Maria-Rosa Sarrias, Francesc E. Borràs

**Affiliations:** 1 REMAR-IVECAT Group, Health Science Research Institute Germans Trias i Pujol, Can Ruti Campus, Badalona, Spain; 2 Department of Cell Biology, Physiology and Immunology, Universitat Autònoma de Barcelona, Bellaterra, Spain; 3 Nephrology Service, Germans Trias i Pujol University Hospital, Badalona, Spain; 4 Innate Immunity Group, Health Sciences Research Institute Germans Trias i Pujol, Badalona, Spain; 5 Network for Biomedical Research in Hepatic and Digestive Diseases (CIBERehd), Badalona, Spain

## Abstract

TLR sensing of pathogens triggers monocyte activation to initiate the host innate immune response to infection. Monocytes can dynamically adapt to different TLR agonists inducing different patterns of inflammatory response, and the sequence of exposure to TLRs can dramatically modulate cell activation. Understanding the interactions between TLR signalling that lead to synergy, priming and tolerance to TLR agonists may help explain how prior infections and inflammatory conditioning can regulate the innate immune response to subsequent infections. Our goal was to investigate the role of MyD88-independent/dependent TLR priming on modulating the monocyte response to LPS exposure. We stimulated human blood monocytes with agonists for TLR4 (LPS), TLR3 (poly(I:C)) and TLR7/8 (R848) and subsequently challenged them to low doses of endotoxin. The different TLR agonists promoted distinct inflammatory signatures in monocytes. Upon subsequent LPS challenge, LPS- and R848-primed monocytes did not enhance the previous response, whereas poly(I:C)-primed monocytes exhibited a significant inflammatory response concomitant with a sharp reduction on cell viability. Our results show that TLR3-primed monocytes are prompted to cell death by apoptosis in the presence of low endotoxin levels, concurrent with the production of high levels of TNFα and IL6. Of note, blocking of TNFR I/II in those monocytes did reduce TNFα production but did not abrogate cell death. Instead, direct signalling through TLR4 was responsible of such effect. Collectively, our study provides new insights on the effects of cross-priming and synergism between TLR3 and TLR4, identifying the selective induction of apoptosis as a strategy for TLR-mediated host innate response.

## Introduction

Monocytes, as key actors of the innate immunity, are equipped with a wide range of pathogen recognition receptors (PRRs) to initiate host defence responses against invading pathogens. Among PRRs, specific Toll-like receptors (TLRs) located in the cell membrane and endosomal compartments sense different components of microorganisms known as pathogen-associated molecular patterns^1^. Activation of TLRs triggers direct antimicrobial activity together with distinct inflammatory patterns depending on the TLR agonist encountered, which modulates the posterior adaptive immune response. Upon agonist engagement, TLRs can follow two main signalling pathways depending on the adaptor molecule recruited to the TIR domains of the TLRs that initiates the intracellular cascade^2^. The myeloid differentiation factor 88 (MyD88)-dependent pathway, which is used by all TLRs except for TLR3, leads to early activation of NFκB for inflammatory cytokine production, specially TNFα by monocytes/macrophages. Instead, viral dsRNA-sensing TLR3, and also TLR4, use the MyD88-independent signalling through the Toll-IL1 domain-containing adaptor inducing IFN-β (TRIF), which activates IRF3 and IRF7 to produce type-I IFN as an antiviral innate response^3,4^. Therefore, gram-negative bacterial lipopolysaccharide (LPS)-sensing TLR4 is the only TLR that has been described to signal through both pathways. At the same time, it is still unclear whether the profiles of TLR responses are exclusively determined by the adaptor molecule they use, as the two main signalling pathways can interact and yield more complex phenotypes.

Monocytes can adapt to infection, and inflammatory conditioning can lead to transient memory states. For instance, continuous TLR4 activation with repeated exposure to bacterial LPS, such as in sepsis, promotes hyporesponsiveness of monocytes to subsequent LPS challenge, a phenomenon termed endotoxin tolerance^5–7^. Moreover, previous studies have shown that different TLR agonists interplay to modulate the inflammatory response to each other^4,8–10^, synergizing to enhance the immune response or promoting heterotolerance to restrain inflammation instead. Understanding the interactions between TLR signalling that lead to synergy, priming and tolerance to TLR agonists may help explain monocyte plasticity and how prior infections and inflammatory conditioning can modulate the innate immune response to secondary infections or co-infections.

We studied the effect of TLR priming of TLR4 (LPS), MyD88-dependent TLR7/8 (using synthetic analogue for single-stranded RNA R848), and MyD88-independent TLR3 (using synthetic analogue for double-stranded RNA poly(I:C)) on the inflammatory response of monocytes to subsequent challenge of TLR4. Our results show that the sequential stimulation of TLR3 and TLR4 leads to the exacerbation of the inflammatory response of monocytes together with the triggering of cell death, which is directly dependent on TLR4 signalling but independent from TNFα.

## Results

### TLR agonists lead to distinct monocyte response signatures

**Fig. 1:**
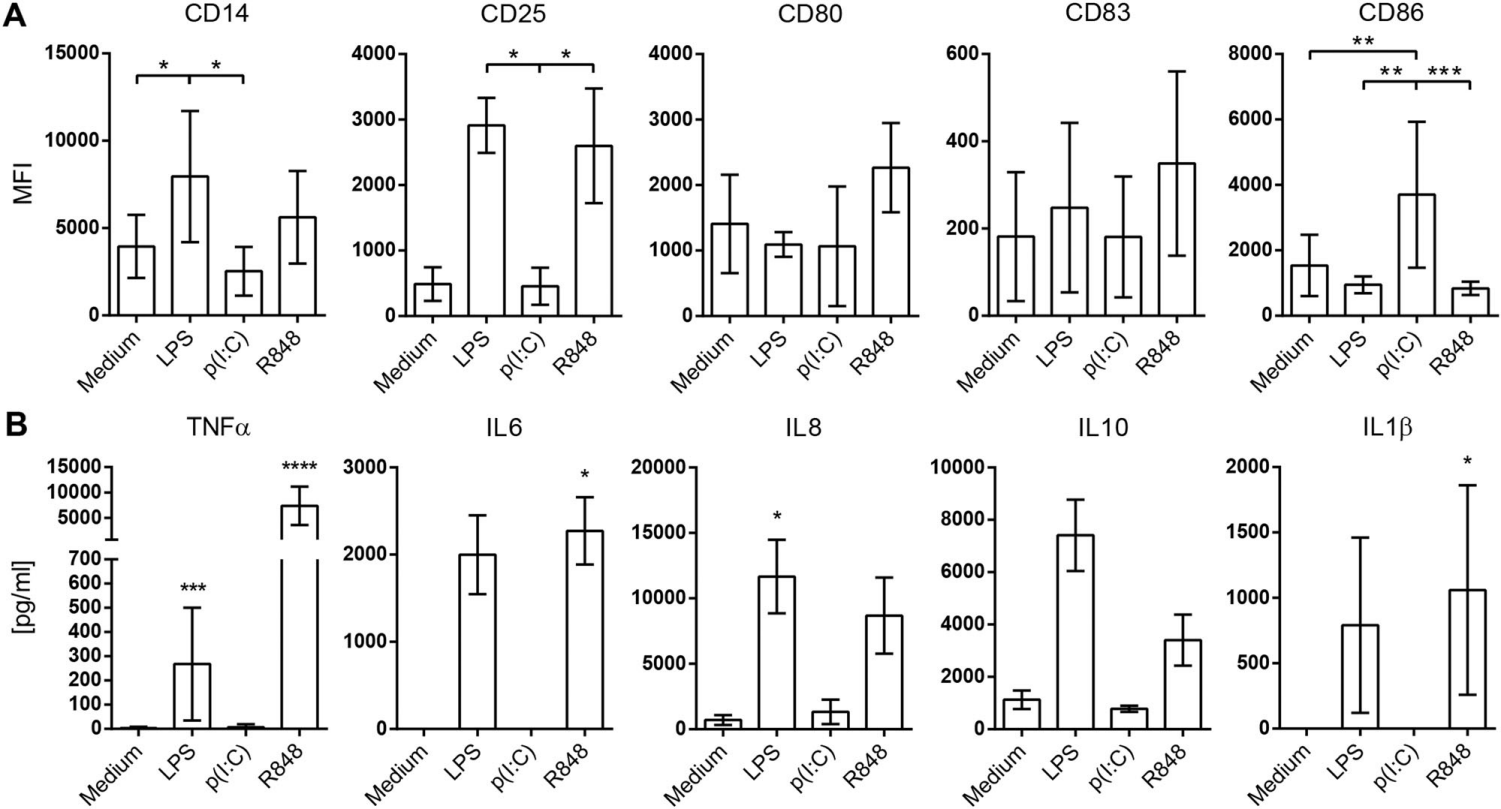
TLR agonists lead to distinct monocyte response signatures. Monocytes were stimulated for 24 h with ligands for TLR4 (LPS), -3 (poly(I:C)) or -7/8 (R848) and checked for cell activation. **a** Monocytes (distinguished by FSC-A/SSC-A) were analyzed for CD14, CD25, CD80, CD83 and CD86 expression by flow cytometry and the MFI values of monocytes are shown. The data are mean ± SD and accounts for more than six independent experiments. **b** TNFα, IL6, IL8, IL10 and IL1β levels found in culture supernatants of monocytes cultured with TLR agonists for 24 h. The data are mean ± SD and accounts for more than four independent experiments. *p < 0.05; **p < 0.01; ***p < 0.001; ****p < 0.0001 by Kruskall–Wallis with Dunn’s post-hoc test

Monocytes were cultured for 24 h in non-stimulated conditions or stimulated with TLR agonists LPS (TLR4, 100 ng/ml), poly(I:C) (TLR3, 50 µg/ml) or R848 (TLR7/8, 2.5 µM). While all TLR ligands prompted cell activation (Fig. 1a, b), the activation signature was different depending on the TLR ligand used for monocyte stimulation. LPS induced an increased expression of CD14 and CD25, while poly(I:C) induced a marked upregulation of CD86, and R848 induced specially CD25, and also CD80 and CD83 (Fig. 1a). In terms of cytokine production, MyD88-dependent TLRs resembled each other, as LPS and R848 quickly triggered the secretion of TNFα, IL6, IL8, IL10 and IL10β, while poly(I:C) did not (Fig. 1b).

### TLR3 priming synergizes with low dose TLR4 for an increased inflammatory response by monocytes

**Fig. 2:**
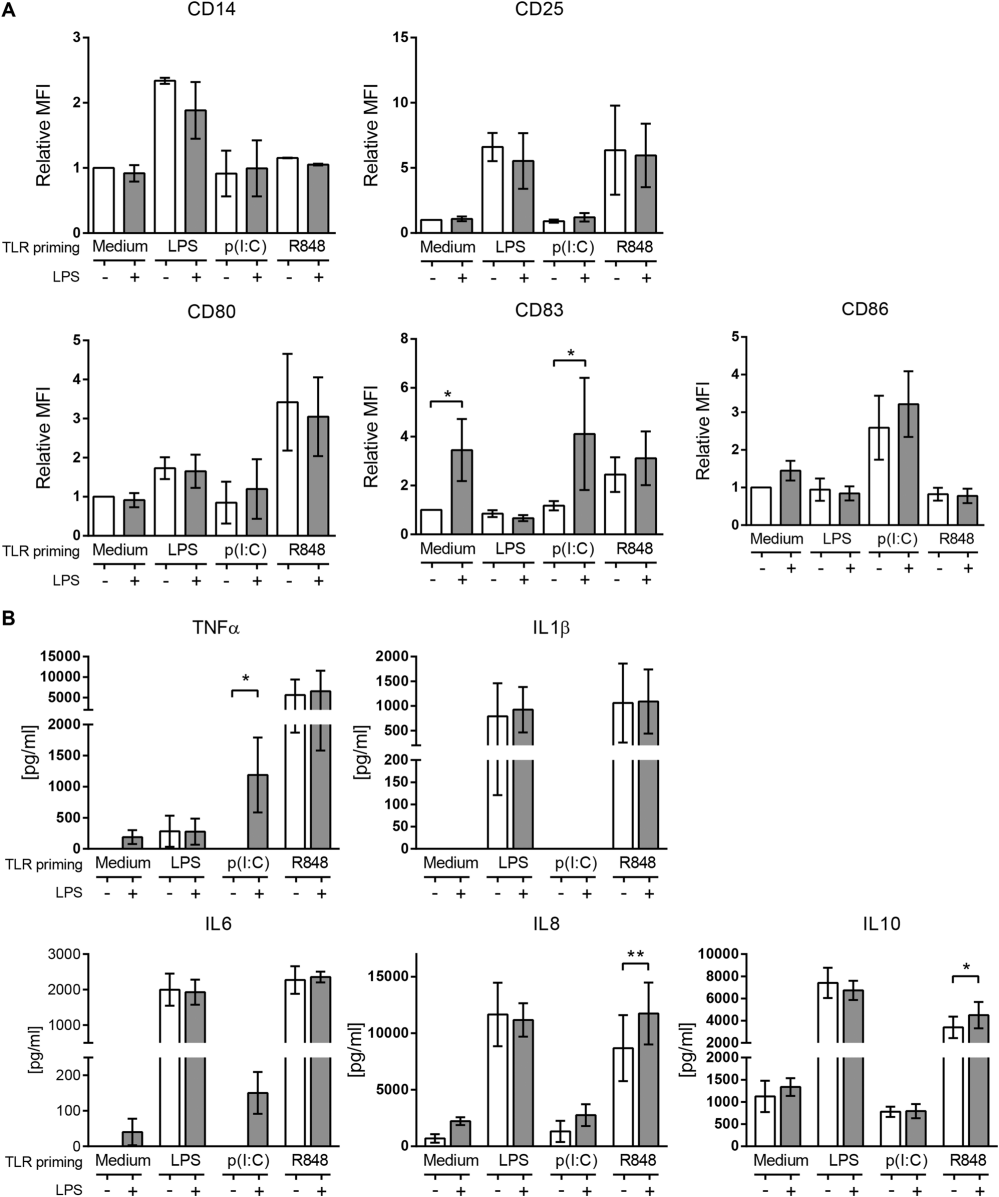
TLR3 priming synergizes with low dose TLR4 for an increased inflammatory response by monocytes. Monocytes were primed or not for 24 h with LPS, poly(I:C) or R848, then challenged with (blue bar) or without (white bar) with 0.2 ng/ml LPS for 5 h. **a** The surface expression of CD14, CD25, CD80, CD83 and CD86 were checked by flow cytometry (monocytes distinguished by FSC-A/SSC-A). The data are mean MFI ± SD relative to each control (non-stimulated monocytes) and accounts for more than three independent experiments. **b** TNFα, IL6, IL8, IL10 and IL1β levels found in culture supernatants of monocytes after LPS challenge. Data is mean ± SD and accounts for four independent experiments. Statistical differences towards LPS response are shown as *p < 0.05; **p < 0.01 by two-way ANOVA

The influence of different TLR agonist priming on modulating a secondary TLR4 response by monocytes was then assessed. After 24 h of culture with a first TLR agonist, monocytes were challenged with a low dose LPS (0.2 ng/ml), and 5 h later, cells were collected to analyze their short-term inflammatory response. Monocytes that had been primed with LPS did not increase their activation markers expression nor their cytokine production after LPS challenge (Fig. 2a, b). R848-primed monocytes were unchanged in terms of activation markers expression but significantly increased their secretion of IL8 and IL10, although they were already produced in great amounts before the LPS challenge (Fig. 2b). On the other hand, monocytes that had been previously stimulated with poly(I:C) boosted their CD83 expression in the same way as non-primed cells (Fig. 2a), and noticeably, only poly(I:C)-primed monocytes exacerbated the pro-inflammatory cytokine production of TNFα and IL6 after the low dose LPS challenge (Fig. 2b). Given the sudden and marked increase on TNFα and IL6, known inducers of inflammation, we sought to study more these cross-primed monocytes.

### Secondary LPS exposure causes cell death to non-primed and TLR3-primed monocytes

**Fig. 3:**
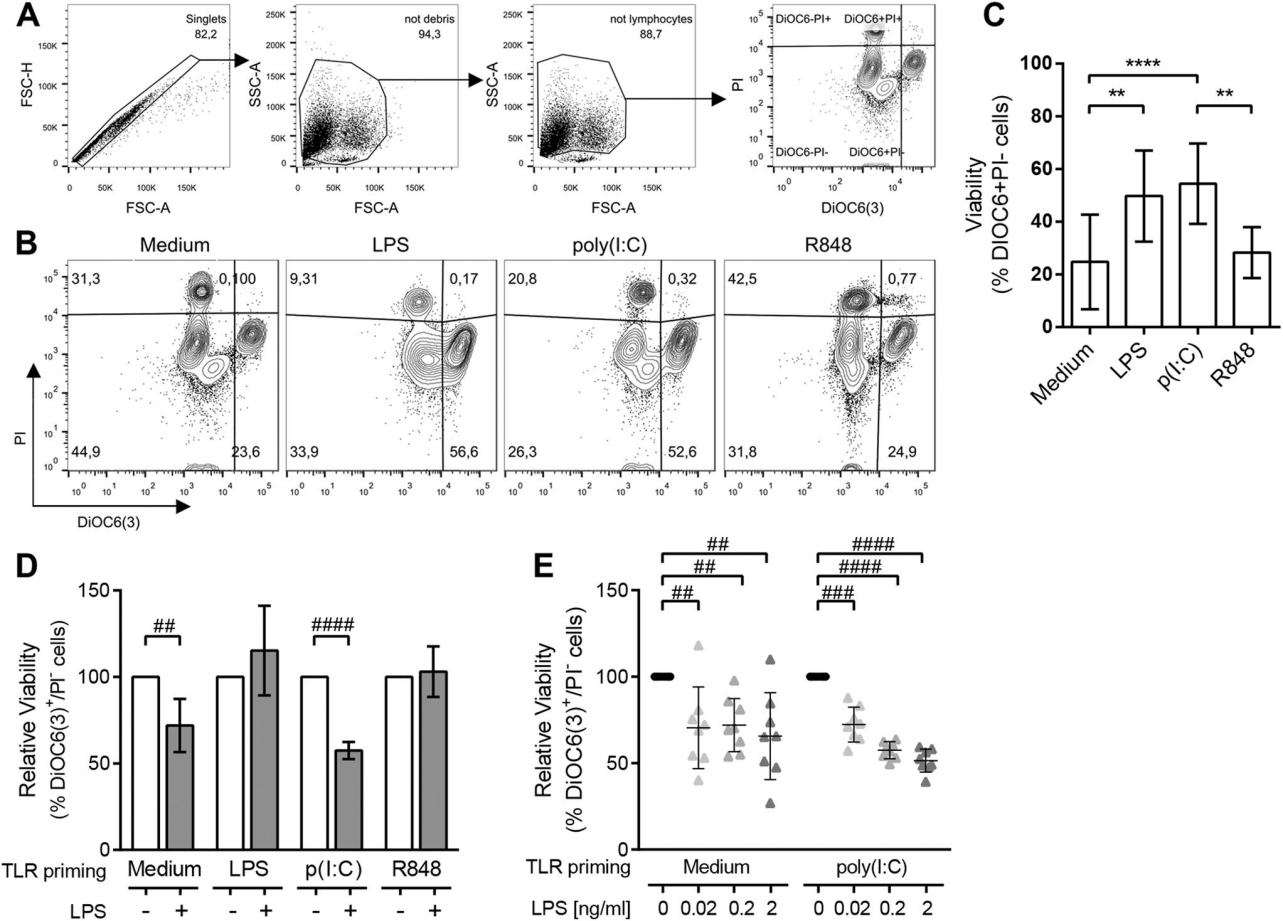
Secondary LPS exposure triggers cell death on TLR3-primed monocytes. **a** Representative plots of the gating strategy used for viability assessment by flow cytometry, where viable cells are recognized as DiOC6(3)^+^/PI^−^, early apoptotic cells lose DiOC6(3) labelling, and both late apoptotic and necrotic cells appear PI^+^. **b**–**c** Representative plots (**b**) and mean viability (**c**) of monocytes after 24 h of TLR activation. The data are mean ± SD of more than nine independent experiments. **d** Monocytes were primed for 24 h with LPS, poly(I:C) or R848, then challenged (blue bars) or not (white bars) with 0.2 ng/ml LPS for 5 h. The data are the mean relative viability ± SD to each control measured as the % of DiOC6(3)^+^/PI^−^ monocytes and accounts for more than four independent experiments. **e** Relative viability measured as the % of DiOC6(3)^+^/PI^−^ cells of non-primed (left) and poly(I:C)-primed (right) monocytes challenged with increasing doses of LPS. Individual experiments are shown and mean ± SD is depicted by horizontal bars. Statistical differences are indicated for **p < 0.01 and ****p < 0.0001 by one-way ANOVA and ##*p* < 0.01; ###*p* < 0.001 and ####*p* < 0.0001 by one-sample *T*-test

To further delineate cellular state and activation, cell viability was monitored by evaluating both mitochondrial function and membrane integrity by flow cytometry. As depicted in Fig. 3a, viable cells were gated according to their functional mitochondrial transmembrane potential (DiOC6(3)^+^) and no permeability to propidium iodide (PI^−^). While non-stimulated monocytes and R848-activated monocytes displayed a reduced viability after 24 h in culture, LPS and poly(I:C) activation rescued monocytes from in vitro cell death (Fig. 3b, c).

We then studied the viability of monocytes after low dose LPS challenge. LPS addition did not modify monocyte viability when the previous stimuli were LPS or R848, but resulted in a reduction in cell viability of non-primed monocytes (−22.4%; Fig. 3d), and importantly on TLR3-primed monocytes (−43.3%). Interestingly, non-primed monocytes heterogeneously responded to LPS-induced cell death (Fig. 3e, left), but poly(I:C) priming consistently committed monocytes from different donors to cell death in a dose-dependent response to low dose LPS challenge (Fig. 3e, right). Even extremely low endotoxin levels (0.02 ng/ml LPS) were already able to induce a 27.6% of cell death, and higher concentrations (0.2 and 2 ng/ml LPS) both triggered the death of around the half of the monocyte population (43.3% and 45.4%, respectively). On the contrary, when the combination of TLR agonists was inverted, so LPS-primed monocytes were stimulated with poly(I:C), it did not lead to cell death nor increased cell activation (data not shown).

**Fig. 4:**
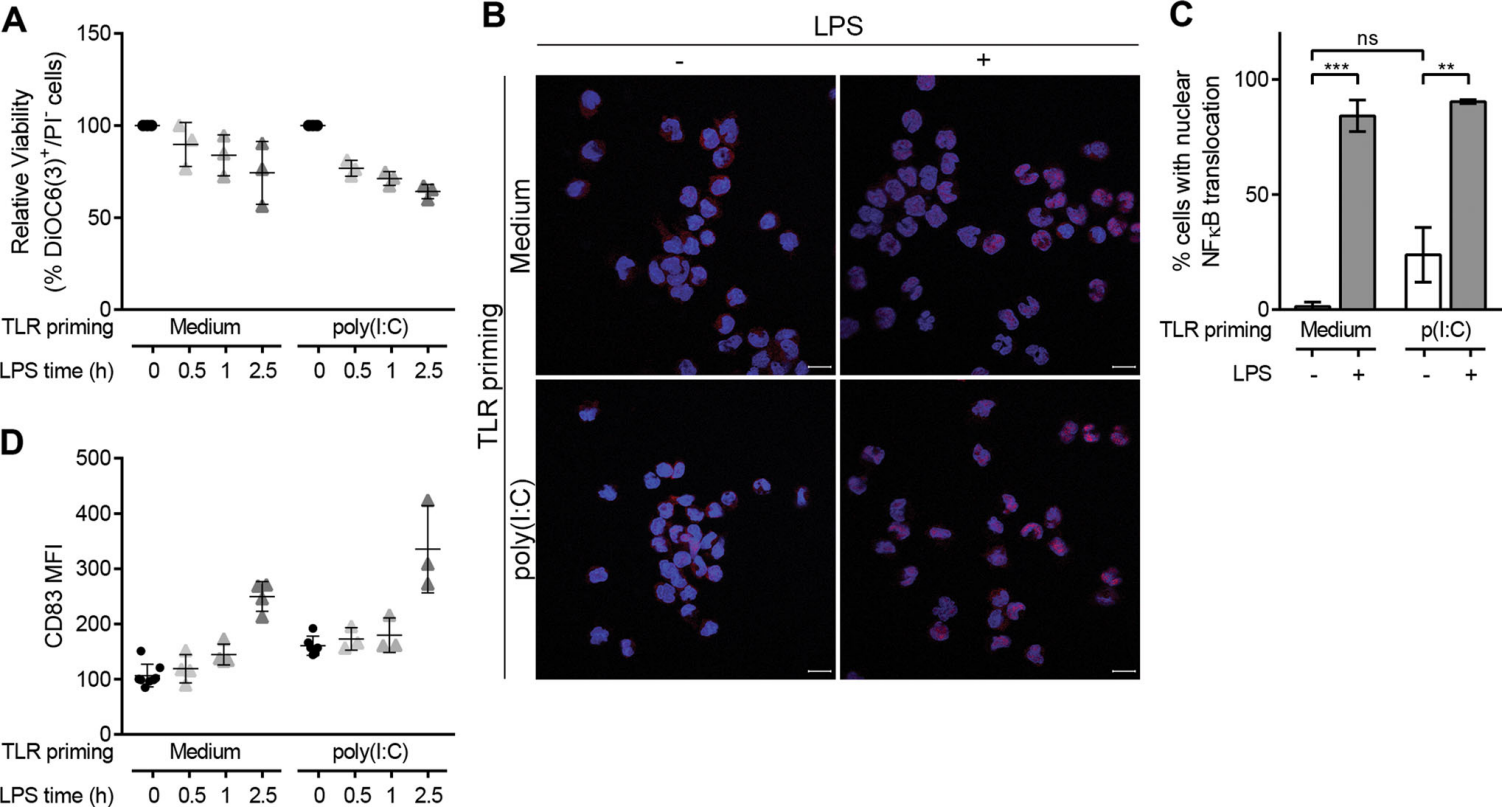
Cell death and cell activation concur in monocytes after low dose LPS stimulation. Non-primed or poly(I:C)-primed monocytes were stimulated with low dose LPS (0.2 ng/ml). **a** Viability was analyzed after 0.5, 1 and 2.5 h. Individual experiments are shown and mean ± SD is depicted by horizontal bars. **b**–**c** NFκB activation was assessed by quantifying cells with RELA nuclear translocation after 30 min from LPS stimulation. **b** Representative images of treated monocytes stained with RELA (red), and nucleus was counterstained with DAPI (blue). RELA can be seen translocated in the nucleus of cells with LPS stimulation. Scale bar = 10 µm. **c** Quantification of cells with RELA nuclear translocation. The data are mean ± SD of two independent experiments. Statistical differences are indicated for **p < 0.01 and ***p < 0.001 by two-way ANOVA. **d** CD83 surface expression of non/poly(I:C)-primed monocytes was analyzed after 0.5, 1 and 2.5 h of LPS stimulation. Individual experiments are shown and mean ± SD is depicted by horizontal bars

A kinetics study with shorter time points showed the rapid and consistent decrease in poly(I:C)-primed monocyte viability after LPS stimulation (Fig. 4a). In an attempt to distinguish whether cell death or cell activation was triggered first, we analysed NFκB activation by quantifying RELA translocation, 30 min after LPS stimulation of non-primed and poly(I:C)-primed monocytes. A 23.8% ± 11.8% of non-stimulated, poly(I:C)-primed monocytes had nuclear RELA (Fig. 4b, c), indicative of NFκB activation after 24 h of poly(I:C) addition. After 30 min of LPS addition (0.2 ng/ml), NFκB activation occurred in the vast majority of cells regardless of the priming state of monocytes (84.2% ± 6.8% non-primed and 90.4% ± 0.8% poly(I:C)-primed). NFκB activation also correlated with an early upregulation of CD83 surface expression (Fig. 4d), the marker that was previously shown to be increased at 5 h post-LPS addition (Fig. 2a). Altogether, these results seem to indicate that cell activation and cell death happen concomitantly.

### LPS triggers apoptosis on TLR3-primed monocytes

**Fig. 5:**
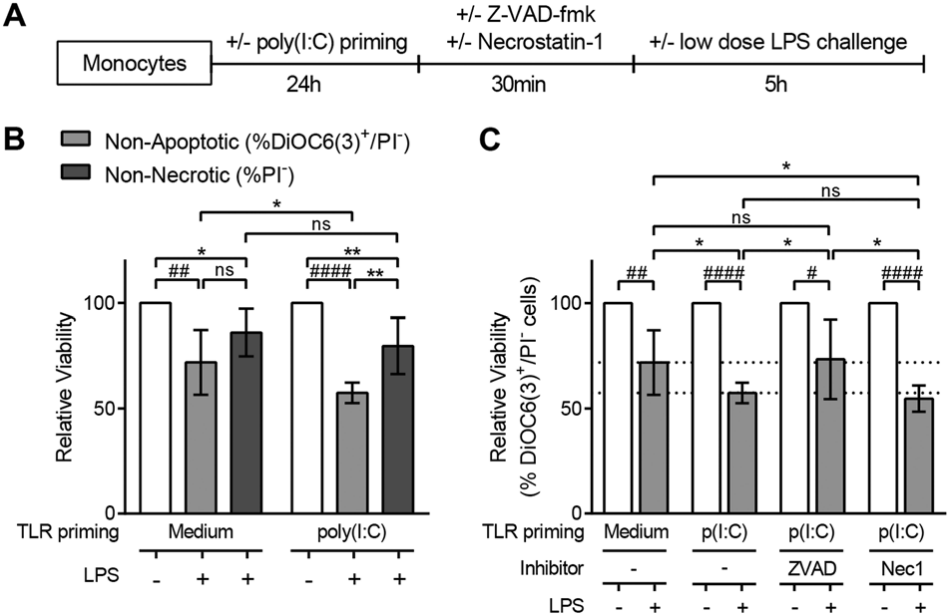
LPS triggers apoptosis on TLR3-primed monocytes. **a** Schematics of the experimental design followed. **b** Relative viability of non-primed (left) or poly(I:C)-primed (right) monocytes after 0.2 ng/ml LPS challenge, measured either as the % of non-apoptotic cells (DiOC6(3)^+^/PI^−^) or non-necrotic cells (PI-). **c** Relative viability (%DiOC6(3)^+^/PI^−^) of non-primed or poly(I:C)-primed monocytes with or without pre-incubation with the pan-caspases inhibitor Z-VAD-fmk (50 µM; *n* = 8) or the RIP1 inhibitor Necrostatin-1 (Nec-1; 90 µM; *n* = 5). The data are expressed as mean ± SD, relative to each control, and accounts for more than four independent experiments. Statistical differences are indicated for *p < 0.05 by two-way ANOVA and #*p* < 0.05; ##*p* < 0.01; ####*p* < 0.0001 by one-sample *T-*test

Given this profound effect on cell viability, we next explored which type of cell death was triggered by low dose LPS on poly(I:C)-primed monocytes (Fig. 5a). The descent in the percentage of viable cells according to only PI labelling was similar for both non- and poly(I:C)-primed monocytes (Fig. 5b), suggesting that necrosis was not fostered. The difference between the two priming conditions was actually found when the monocytes’ mitochondrial function was analyzed. An increase in cell death according to DiOC6(3) staining in poly(I:C)-primed monocytes compared to non-primed monocytes was observed already at 5 h after LPS addition (Fig. 5b).

As these results were suggestive of apoptosis induction, we next examined the effect of the pan-caspases inhibitor Z-VAD-fmk on the viability of monocytes. The addition of Z-VAD-fmk prevented the cell death fostered by poly(I:C) priming of monocytes after sequential LPS exposure (Fig. 5c), indicating thus an apoptotic cell death of monocytes sequentially activated with poly(I:C) and LPS.

To further discard caspase-independent types of programmed cell death, necrostatin-1 (Nec-1) was added to cell culture. Programmed necrosis or necroptosis has been identified also in monocytes^11,12^. Necroptosis signalling involves the activation of the kinase domain of receptor-interacting protein 1 (RIP1), which can be specifically inhibited with Nec-1^13^. Our results showed that poly(I:C)/LPS-induced cell death was not prevented when cells were pre-incubated with Nec-1 (Fig. 5c), discarding a necroptotic cell death. Moreover, pyroptosis could also be ruled out as IL1β—a crucial cytokine in this process—was not produced by poly(I:C)/LPS-stimulated monocytes (Fig. 2b).

### LPS promotes apoptosis independently of TNFα production but downstream of TLR4 signalling in TLR3-primed monocytes

**Fig. 6:**
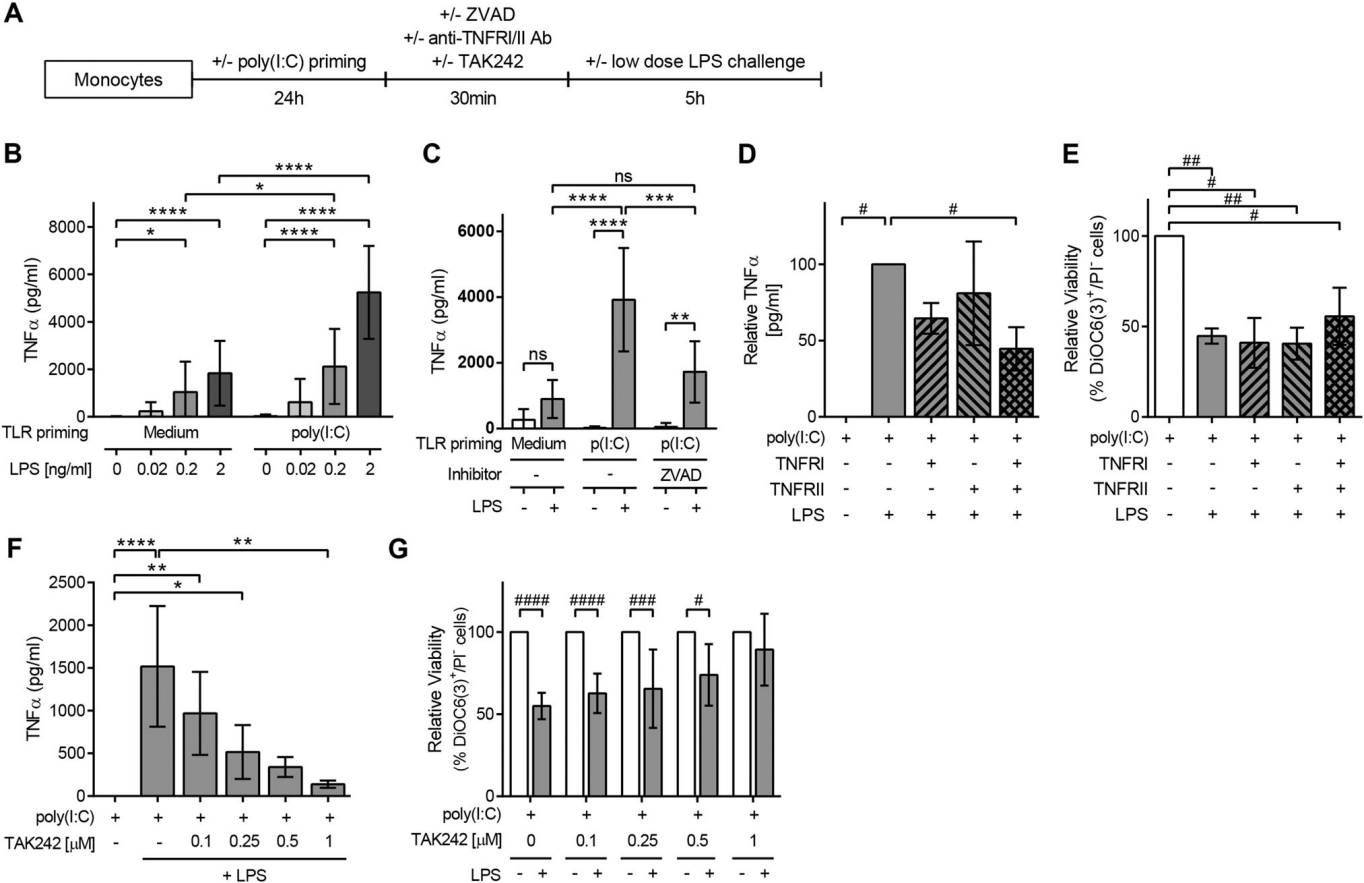
LPS triggers apoptosis independently of TNFα production but downstream of TLR4 signalling in TLR3-primed monocytes. **a** Schematics of the experimental design followed. **b** TNFα levels found in culture supernatants of non-primed (left) and poly(I:C)-primed (right) monocytes challenged with increasing doses of LPS. The data are mean ± SD of nine independent experiments. **c** TNFα levels produced by non-primed or poly(I:C)-primed monocytes with or without pre-incubation with Z-VAD-fmk (50 µM) after 5 h of LPS stimulation. The data are mean ± SD of six independent experiments. *p < 0.05; **p < 0.01; ***p < 0.001 and ****p < 0.0001 by two-way ANOVA. **d**–**e** Poly(I:C)-primed monocytes were pre-incubated with neutralizing antibodies for TNFRI/II (*n* = 3) and then challenged with 0.2 ng/ml LPS. TNFα levels (**d**) and monocytes’ relative viability (**e**) were checked 5 h later. **f**–**g** Poly(I:C)-primed monocytes were pre-incubated with the TLR4 signalling inhibitor TAK242 (*n* = 7) and then challenged with 0.2 ng/ml LPS. The TNFα levels (**f**) and the relative viability of cells (**g**) are depicted as mean ± SD. *p < 0.05; **p < 0.01 and ****p < 0.0001 by Kruskall–Wallis with Dunn’s post-hoc test and #*p* < 0.05; ##*p* < 0.01; ###*p* < 0.001 and ####*p* < 0.0001 by one-sample *T-*test

We next studied possible inducers of the apoptosis promoted by LPS in TLR3-primed monocytes (Fig. 6a). One of the classical triggers of apoptosis is the signalling through TNF receptors. While TNFα was not produced in poly(I:C)-stimulated monocytes (Fig. 2b), these cells produced high levels of TNFα in a dose-dependent manner upon LPS challenge (Fig. 6b). TNFα production was enhanced by two-fold by poly(I:C) priming compared to non-primed cells (Fig. 6b). The levels of TNFα produced were as low as non-primed cells when apoptosis was blocked by treating poly(I:C)-primed monocytes with Z-VAD-fmk before LPS addition (Fig. 6c). The addition of neutralizing antibodies for TNFRI and TNFRII diminished the production of TNFα by half when both were used (Fig. 6d), but could not inhibit the death induced by LPS challenge to poly(I:C)-primed monocytes (Fig. 6e).

Noticeably, TNFα production was abrogated by blocking specifically TLR4 signalling with TAK242 (Fig. 6f). The use of this TLR4 antagonist directly correlated with the complete inhibition of LPS-induced cell death (Fig. 6g). All together, these results suggested the direct association between TLR4 ligation and signalling with the induction of cell death by LPS, and ruled out a TNFR-dependent induction of apoptosis in TLR3-primed monocytes.

**Fig. 7:**
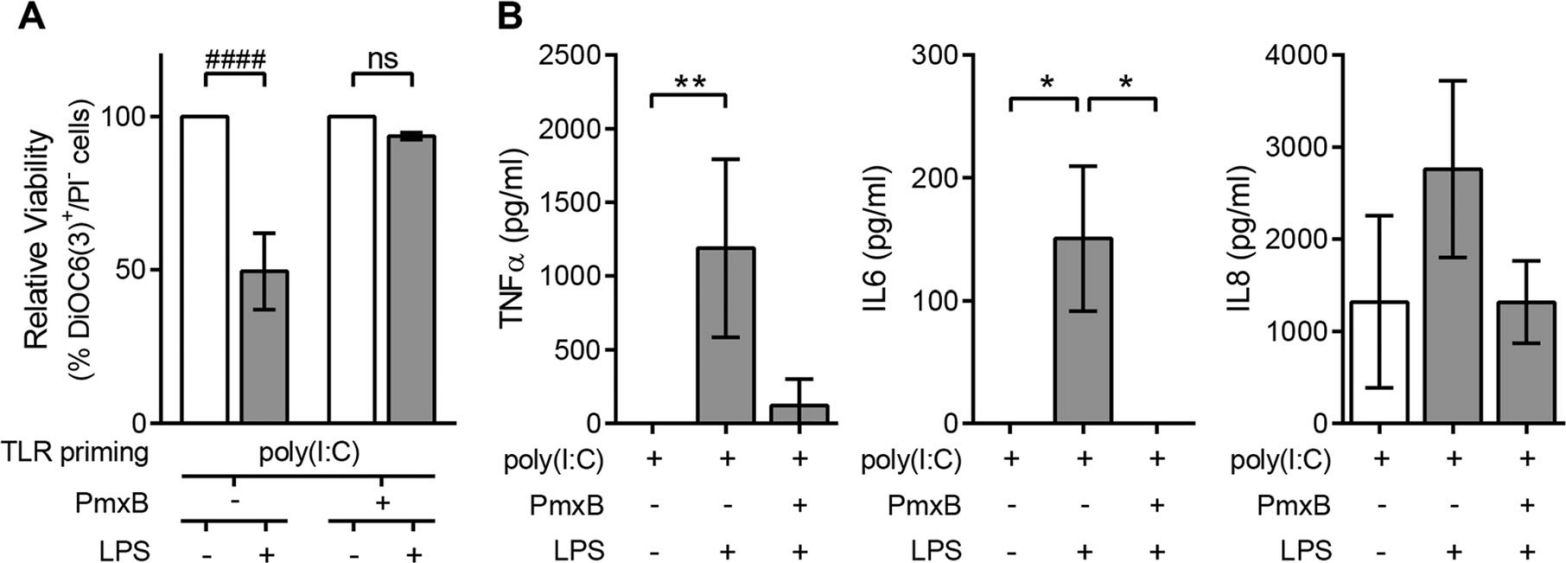
PmxB abrogates LPS-mediated apoptosis and cytokine production in TLR3-primed monocytes. PmxB was mixed with LPS before its addition to poly(I:C)-primed monocytes. **a** Relative viability of monocytes after 5 h of LPS challenge. **b** Inflammatory cytokine levels (TNFα, IL6 and IL8) found in culture supernatants of poly(I:C)-primed monocytes challenged with LPS or LPS pre-incubated with PmxB. The data are mean ± SD of three independent experiments. ####*p* < 0.0001 by one-sample *T-*test; *p < 0.05 and **p < 0.01 by Kruskall–Wallis with Dunn’s post-hoc test

Further insight on the central role of LPS ligation in the TLR3-primed monocyte-induced cell death and activation was obtained when blocking LPS traces with polymyxin B (PmxB). PmxB was simply mixed with LPS before its addition to monocytes, and while it was innocuous by itself, it successfully inhibited the LPS-induced cell death (Fig. 7a), and also the inflammatory cytokine production of TNFα, IL6 and IL8 in TLR3-primed monocytes (Fig. 7b).

## Discussion

The present study reports that TLR3 priming boosted the inflammatory response of human monocytes to subsequent low dose LPS challenge, with a concomitant and drastic apoptotic cell death of half the monocyte population.

**Fig. 8:**
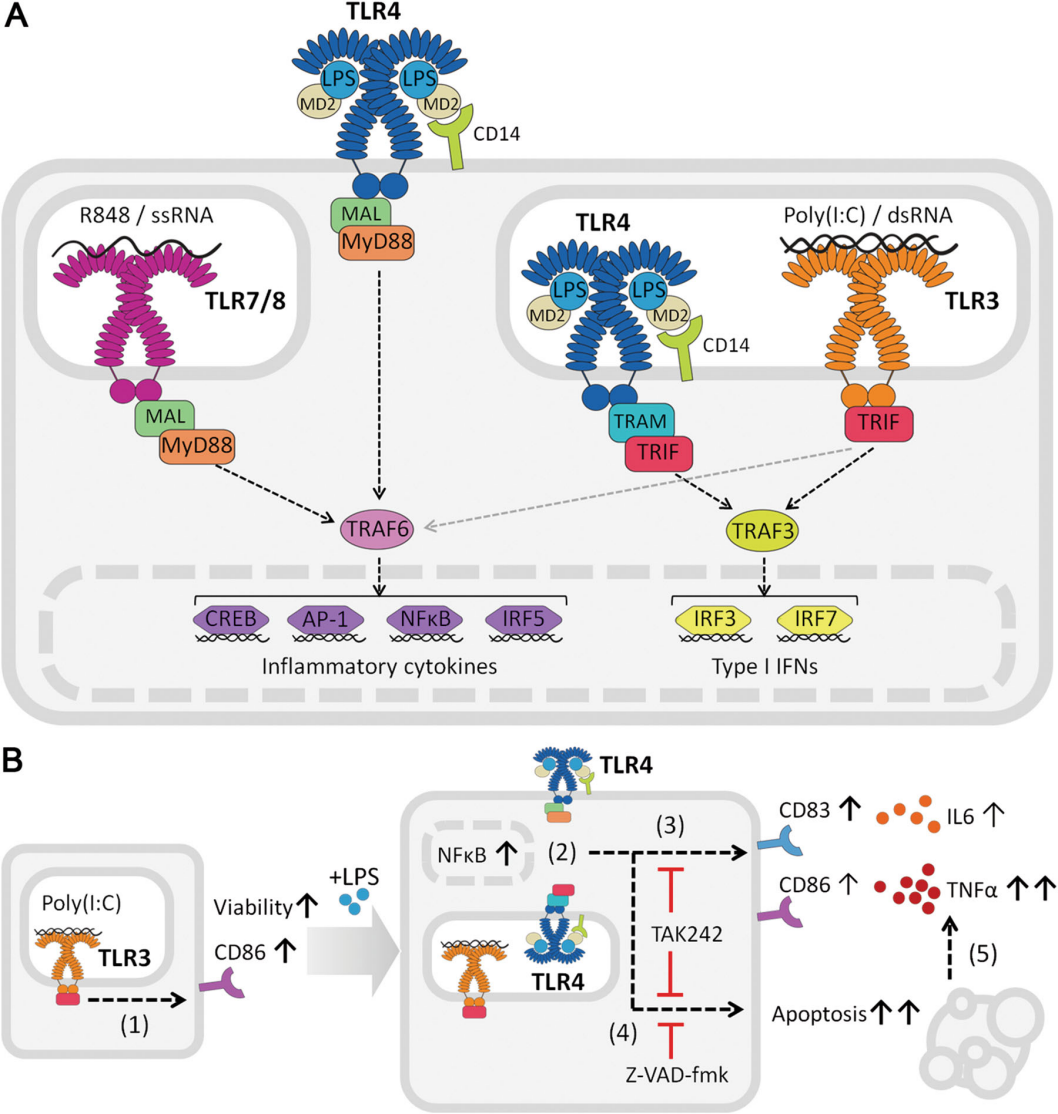
Schematic representation of the TLR signalling pathways and results. **a** Overview of the signalling pathways involved in the TLRs studied. Upon R848 or ssRNA ligation, TLR7/8 uses the adaptor molecule MyD88 to signal through TRAF6 and activate the transcription factors inducing inflammatory cytokine production. Poly(I:C) or dsRNA bind to TLR3, that uses the adaptor molecule TRIF to activate TRAF3 and produce type I IFNs to initiate an antiviral immune response. It can also activate TRAF6. TLR4, recognizing LPS with the help of MD2 and CD14, is the only TLR that can signal through both MyD88 and TRIF. **b** Stimulation of monocytes with poly(I:C) increased their viability and expression of CD86 (1). When poly(I:C)-primed monocytes were then stimulated with low doses of LPS, NFκB signalling was activated and translocated to the nucleus (2), they upregulated CD83 expression, and boosted TNFα and IL6 production (3), concomitant with death by apoptosis (blocked by Z-VAD-fmk) of half the population (4). These effects were inhibited by TAK242, indicating the dependence on direct signalling through TLR4. The induced cell death caused the increased TNFα production (5)

Studies before have shown the interplay between TLR signalling pathways, leading to distinct transcriptional and thus phenotypic profiles that might be not simply classified as MyD88-dependent or independent^14^. For instance, TLR3 has been reported to indirectly activate NFκB^1,15,16^, as we have also observed. Nevertheless, the classification and study of TLR responses according to their intracellular signalling adaptors greatly defines monocyte response to TLR ligation, as we described here and others have observed^1,3,4,17^. In our hands, monocytes stimulated with agonists for MyD88-dependent TLRs (LPS-TLR4 and R848-TLR7/8, summarized in Fig. 8a) showed identical profiles of inflammatory cytokine secretion, while the activation phenotype revealed some specific features depending on the TLR agonist used. However, TLR3 priming did not produce inflammatory cytokine secretion. The observed absence of inflammatory cytokine production after single TLR3 stimulation has been observed before^4,8^, and it could be explained by the single signalling of TRIF towards IRF3/7 activation, with a minor TRAF6 co-activation of NFκB (Fig. 8a). At the same time, timing of TLR responses could be different between the two signalling pathways. Yet, poly(I:C) promoted monocyte activation and priming, shown by the CD86 upregulation, NFκB nuclear translocation, increased survival compared to control conditions and exacerbated response to secondary low dose LPS stimulation.

At the same time, recognition of viral RNA is not exclusive of TLR3, as it has been described before to be recognised by the cytoplasmic RNA helicases, retinoic acid inducible gene-I (RIG-I), that binds to short dsRNA (<1000 bp) and 5′-triphosphate single-stranded RNA (5′-PPP-RNA), and melanoma differentiation-associated gene 5 (MDA-5), which detects long dsRNA (>1000 bp)^18–20^. The data on human monocyte-derived macrophages indicates that they do express MDA-5 but lack RIG-I^21^. High-molecular weight poly(I:C), which is the one used in our experiments, has been shown to be detected preferentially by TLR3 when added to the culture medium, while it needs to be actively delivered to the cytosol to promote MDA-5 detection by means of transfection^20,22,23^, and only after shortening to further signal through RIG-I^18^. Furthermore, response to poly(I:C) greatly differs when detected by TLR3 compared to MDA-5, given that great amounts of IFNβ and TNFα are produced after transfection of poly(I:C) and thus detected by MDA-5, but not when it is added to the culture medium and detected instead by TLR3^24^. Therefore, in our case, as poly(I:C) is simply added to the culture medium and no TNFα was detected after its addition, it all would indicate TLR3 ligation rather than signalling through MDA-5 is happening.

The heightened response to sequential LPS exposure is particularly relevant given the low doses of LPS used. These observations can have implications in the pathological context, and may also be significant in vitro. In this sense, recombinant proteins and serum-derived products can contain minimal amounts of LPS, which even referred as “endotoxin-free,” could be altering results by activating monocytes. In fact, previous studies showed already how these same very low doses of LPS (as little as 0.2 EU/ml) can induce inflammatory cytokine production by human monocytes and dendritic cells^25^. As we show in the present study, adding PmxB to the reagent of choice could be an efficient and easy precautious method to avoid experiment artefacts.

Monocyte response to combined TLR ligation can result both in synergy and tolerance, yielding induction or restriction of inflammation respectively, to fine-tune the innate and adaptive immune responses. This equilibrium has actually been the focus of study before, especially in the context of sepsis, in which endotoxin tolerance is a hallmark of this disease^6,7,26^. “Endotoxin tolerance” refers to the refractory state to LPS challenge that innate cells, including monocytes, have after repeated LPS exposure. Both the timing and the dose of LPS were proven to be crucial to induce either priming or tolerance of TLR4 response. Yuan et al.^9^ showed how high doses of LPS were responsible of hyporesponsiveness to LPS challenge, but low LPS doses induced a low-level inflammatory state on murine macrophages. Our results suggest that monocytes do not suffer major changes after repeated LPS exposure, as they kept on expressing activation markers and producing high amounts of inflammatory cytokines, probably due to insignificant change on overall LPS concentration.

When using combinations of different TLRs, temporal and sequential TLR activation may also produce synergy or tolerance. For instance, Napolitani et al. showed maximal synergy between TLR4 and TLR8 in human monocyte-derived dendritic cells when they were given in a 4-hour window regardless of the order, while TLR3 and TLR8 response was enhanced uniquely when poly(I:C) was added before R848 and not otherwise^27^. Bagchi and colleagues combined MyD88-dependent and independent TLR agonists and while some combinations yielded cross-tolerance, they found that poly(I:C) priming and secondary TLR2 (MyD88-dependent) or TNF activation promoted cross-priming of murine macrophages^4^, although only the cytokine profile was analysed.

In line with these reports, we describe how the sequential exposure of human monocytes to TRIF- (TLR3) and TRIF/MyD88-dependent (TLR4) agonists resulted in the augmented inflammatory response to the second agonist, leading to the cross-priming of monocytes towards a drastic activation of cells, while the inverted combination of TLR agonists did not. Although TLR3 is designed to mount an antiviral immune response after sensing dsRNA (a replication intermediate of several virus), and TLR4 would promote cell activation upon gram-negative bacteria detection, synergy between TLR3 and TLR4 has been proven before to successfully cooperate against infection. For instance, systemic activation of TLR3/TRIF promotes resolution of gram-negative gut infection^28^, while antiviral response is worse in TLR4 knockout mice^29^. Also, TNFα production—which quickly spiked after the sequential TLR3–TLR4 activation of monocytes—contributes to virus clearance in the first stages of a viral infection^30^.

The effect of TLR signalling on monocyte biology is normally focused on the activation response of cells, while viability of cells is often overlooked. In our experiments, TLR4-activation of TLR3-primed monocytes was concomitant with the apoptotic cell death of half the population (as summarized in Fig. 8b) as determined by mitochondrial membrane function, a highly sensitive and stringent method to measure early apoptotic cell death^31–33^.

TLR3-primed monocytes apoptotic cell death was most probably due to the activity of pre-mitochondrial caspases given that pan-caspases inhibitors were able to abrogate the loss of mitochondrial potential. Apoptosis has been shown before to be induced directly through LPS-TLR4 signalling^34,35^. As little as 1 ng/ml has been demonstrated to trigger necrotic and apoptotic monocyte death^36^ and intrinsic and extrinsic apoptosis in the monocytic cell line THP-1^37^. Indeed, Liu and colleagues demonstrated the involvement of the mitochondrial apoptotic pathway in LPS-related death, although in their model apoptosis could be prevented by blocking TNFR. In our case, TNFα production spiked after LPS challenge in TLR3-primed human peripheral blood monocytes, caused by apoptosis of cells, and blockade of TNFR ligation did not rescue cells from death. Instead, monocyte death was depending on direct triggering of TLR4 signalling cascade as proven by direct and specific blockage with TAK242^38–40^. These results seem to discard the involvement of other than TLR4 for LPS detection, such as cytoplasmic PRRs^41^. Similarly, the absence of IL1β and the fact that Nec-1 did not prevent cell death ruled out the induction of, pyroptosis^42,43^ and necroptosis^44^, respectively.

In the context of infection, apoptotic cell death is a well known antiviral mechanism for the host to eliminate virus-infected cells. The combination of the boosted inflammatory cytokine production with the TLR4-dependent death that we describe may enhance the antiviral response of host monocytes by triggering an inflammatory immune response and rapidly eliminating infected cells to stop virus spread^45,46^. This secondary activation with LPS could be happening in the case of co-infection of bacteria and virus or also in the presence of sub-clinical pathology. Given the low doses of LPS needed to elicit this effect, residual endotoxin presence would be enough to trigger these effects in the context of viral infection alone. Also, in the immediacy of an inflammatory site, LPS leakage or translocation can occur from a localized infection to the bloodstream, and affect circulating monocytes^47,48^, as in Inflammatory Bowel Disease or Cystic Fibrosis^47,49–51^.

In summary, the sequential collaboration of TLR3 and TLR4 to boost the inflammatory cytokine secretion of human monocytes, concomitant with the drastic triggering of cell death, may be a novel strategy for TLR-mediated host innate response to fight pathogen spread after infection.

## Materials and methods

### Monocyte isolation and culture

The study protocols were approved by the Clinical Research Ethics Committee of our institution (Comitè Ètic d’Investigació Clínica, HuGTiP, Ref. CEIC: PI^−^13-011), and conformed to the principles outlined in the Declaration of Helsinki.

Peripheral blood monocytes were obtained from leucocyte residues from healthy donors provided by the Blood and Tissue Bank (Barcelona, Spain). Succinctly, peripheral blood mononuclear cells were depleted of CD3^+^ cells using the RosetteSep^TM^ Human CD3 Depletion Cocktail (StemCell Technologies, Seattle, WA) during a Ficoll-Paque density centrifugation (GE Healthcare, Uppsala, Sweden) and monocytes were then positively selected by MagniSort™ Human CD14 Positive Selection Kit (eBioscience, San Diego, CA), as recommended by the manufacturer. Monocytes were assessed for purity by CD14-FITC staining (BD, San Jose, CA), obtaining >95% CD14^+^ cells analysed in a Canto II flow cytometer (BD).

Culture medium comprised RPMI 1640 medium supplemented with 100 µg/ml streptomycin, 100 IU/ml penicillin and 5% heat-inactivated human AB serum (H4522, Sigma, St Louis, MO). Cells were counted using PerfectCount Microspheres (Cytognos, Salamanca, Spain) and viability was >94% as determined by 7-AAD exclusion by flow cytometry (FACSCanto II, BD).

### Cell stimulation

Cells were seeded in 96-well flat-bottomed plates at 1 × 10^6^ cells/ml and were first stimulated with TLR agonists: 100 ng/ml LPS (*Escherichia coli* O111:B4; Sigma), 50 µg/ml poly(I:C) (High-molecular weight; Invivogen, San Diego, CA) or 2.5 µM R848 (Alexis Biochemicals, San Diego, CA) as indicated for each experiment. After 24 h, low doses of LPS were added to monocytes (0.02, 0.2 or 2 ng/ml) and supernatant and cells were collected 5 h later, or else as indicated, to assess cytokine secretion, viability and cell activation phenotype.

Alternatively, PmxB (2 µg/ml; Sigma) was mixed with LPS before its addition to monocytes, incubated for 30 min at 37 °C.

### Cytokine measurement

Cell supernatants were collected, cleared of debris by centrifuging at 400 g 5 min and kept at −20 °C. The levels of cytokines were measured by ELISA following the manufacturers’ instructions: IL6, IL8 were purchased from ImmunoTools (Friesoythe, Germany), IL10 and TNFα from U-Cytech (Utrecht, The Netherlands) and IL1β from R&D Systems.

### Cell activation phenotype

Monocytes were stained with fluorochrome-conjugated antibodies CD14-FITC, CD25-PE-Cy5, CD83-APC (BD) and CD80-PEVio770 and CD86-PE (Miltenyi Biotech, Bergisch Gladbach, Germany) for 15 min at room temperature, washed with PBS and analysed in a Canto II flow cytometer (BD).

### Cell death assessment

To assess the type of cell death, cells were pre-incubated with the pan-caspases inhibitors Z-VAD-fmk, Q-VD-oPH (50 µM and 20 µM after titration analysis; Sigma), the RIP1 inhibitor Nec-1 (90 µM; Sigma), the neutralizing antibodies against TNFRI and TNFRII (clones 16803 and 22221; R&D Systems, Abington, UK) or the TLR4 signalling inhibitor TAK242 (Calbiochem, San Diego, CA), which were added 30 min prior to secondary, low dose (0.02, 0.2 or 2 ng/ml) LPS stimulation of monocytes^39,40,52,53^.

After culture, monocytes were detached from wells using cold PBS for 20 min at 4 °C. Cells were then washed and stained with 40 nM of the potentiometric mitochondrial probe 3, 3′-dihexyloxacarbocyanine iodide (DiOC6(3); Invitrogen, Carlsbad, CA) and 5 µg/ml PI (Sigma-Aldrich). After incubation for 1 h at 37 °C, cells were analyzed, without washing steps, in a LSR Fortessa flow cytometer (BD) equipped with FACSDiva software (BD). Debris was excluded from analysis by size and complexity (FSC/SSC) and live cells were then gated as live by DiOC(3)^+^/PI^−^, or exclusively PI^−^, using the FlowJo X software (Tree Star Inc, Ashland, OR).

### NFκB nuclear translocation analysis

Monocytes (10^5^ cells/well) were plated on Millicell EZ slides (Merck Millipore, Darmstadt, Germany) and primed or not with 50 µg/ml poly(I:C) for 24 h, after which cells were stimulated with 0.2 ng/ml LPS for 30 min. They were then washed once with PBS and fixed with PBS containing 4% paraformaldehyde (Panreac) for 30 min at RT. Next, they were incubated with permeabilization and blocking buffer (PB buffer; PBS containing 0.3% Triton X-100, 5% foetal bovine serum (Gibco) and 5% human AB serum (Sigma-Aldrich)) for 1 h at RT. Cells were subsequently incubated with a poAb anti- RELA (Cell Signaling Technology, 8242) overnight at 4 °C in PB buffer (1/1000). Then, cells were incubated with Alexa Fluor 647 F(ab′)2 fragment of goat anti-rabbit IgG (Molecular Probes, A-21246) for 1 h at RT in PB buffer (1/1000). Between steps, unbound antibodies were removed with three washes with PBS. Finally, coverslips were mounted in ProLong Gold Antifade reagent with DAPI (Life Technologies), sealed and left overnight at 4 °C. The slides were examined under an Axio Observer Z1 DUO LSM 710 confocal system (Carl Zeiss Microscopy GmbH, Jena, Germany) and analyzed with ZEN Black software (Carl Zeiss Microscopy GmbH). The amount of cells with RELA nuclear translocation was counted in at least six different fields (>200 cells) per condition from two independent experiments, as described^54,55^.

### Statistical differences

Values are expressed as mean ± standard deviation. The Kolmogorov–Smirnov analysis was used to check for normality of data and appropriate statistical tests are indicated for each dataset. The analyses were performed using the Graphpad Prism (6.0 version) and differences were considered significant when *p* < 0.05. As we are working with leucocyte residues from different human donors, and as these primary cells have an intrinsic high variability in the percentages of live monocytes that can be found in vitro, we normalized the data pair-wise to best read the effects of stimulating primed cells.
